# One-pot synthesis of CdS/CeO_2_ heterojunction nanocomposite with tunable bandgap for the enhanced advanced oxidation process

**DOI:** 10.1038/s41598-023-34742-3

**Published:** 2023-05-12

**Authors:** Vishal Gadore, Soumya Ranjan Mishra, Md. Ahmaruzzaman

**Affiliations:** Department of Chemistry, National Institute of Technology Silchar, Silchar, Assam 788010 India

**Keywords:** Environmental sciences, Nanoscience and technology

## Abstract

Herein, a binary nanocomposite CdS/CeO_2_ has been fabricated via a one-pot co-precipitation method for the degradation of Rose Bengal (RB) dye. The structure, surface morphology, composition, and surface area of the prepared composite were characterized by transmission electron microscopy, scanning electron microscopy, X-ray powder diffraction, X-ray photoelectron spectroscopy, Brunaur–Emmett–Teller analysis UV–Vis diffuse reflectance spectroscopy and photoluminescence spectroscopy. The prepared CdS/CeO_2_(1:1) nanocomposite has a particle size of 8.9 ± 0.3 nm and a surface area of 51.30 m^2^/g. All the tests indicated the agglomeration of CdS nanoparticles over the surface of CeO_2_. The prepared composite showed excellent photocatalytic activity in the presence of hydrogen peroxide under solar irradiation towards the degradation of Rose Bengal. Near to about complete degradation of 190 ppm of RB dye could be achieved within 60 min under optimum conditions. The enhanced photocatalytic activity was attributed to the delayed charge recombination rate and a lower bandgap of the photocatalyst. The degradation process was found to follow pseudo-first-order kinetics with a rate constant of 0.05824 min^−1^. The prepared sample showed excellent stability and reusability and maintained about 87% of the photocatalytic efficiency till the fifth cycle. A plausible mechanism for the degradation of the dye is also presented based on the scavenger experiments.

## Introduction

The industrialization has improved the standard of living. Many industries flourished; credit goes to the industrial revolution^1^. However, rapid and uncontrolled industrialization has polluted our environment in many ways. Pharmaceuticals, textile, paint, and chemical industries flourished, leading to the generation of harmful chemicals. Moreover, due to large-scale industrialization, many pollutants are dumped into water bodies, resulting in water and environmental pollution^2^. When present in wastewater, dyes, and pigment affect the growth of aquatic plants by blocking the sunlight from reaching the surface. They are not degraded easily, resulting in high BOD of water samples. Therefore, it is necessary to develop an environment-friendly material that can harvest sunlight and degrade organic pollutants in water in today's world. Photodegradation is the easiest and most economical way of removing organic compounds from wastewater^3^.

**Figure 1:**
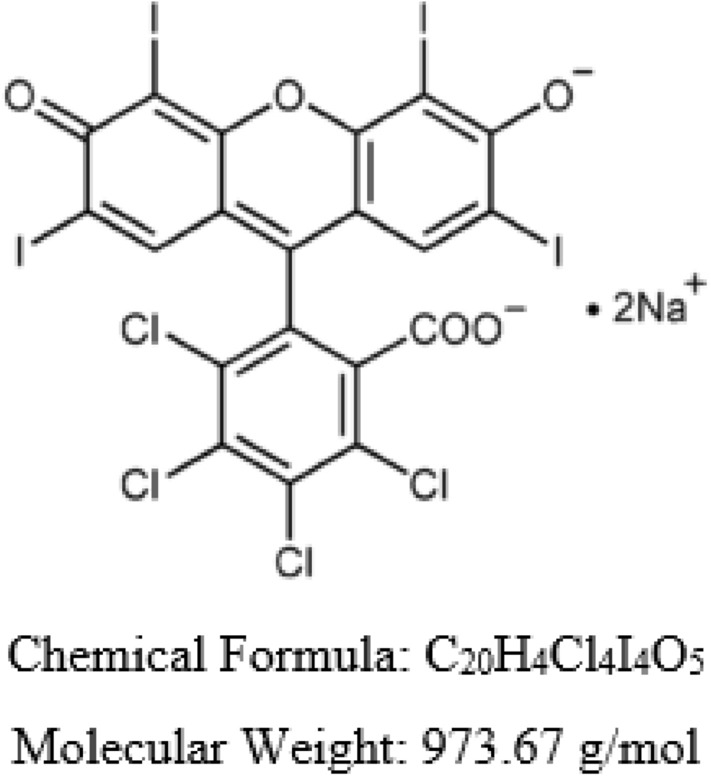
Structure of Rose Bengal dye.

Pollutants such as pesticides, organic dyes, benzene compounds, phenols, and industrial effluents are quite toxic and pose a risk to human well-being and the aquatic ecosystem^4,5^. Phenols result in a foul odour of water. The use of chlorine-based disinfectants results in the formation of chlorinated phenols, which are even more toxic to the ecosystem. Among these pollutants, the removal of dyes has remained a serious concern among researchers, as almost 20% of the dyes are lost during the dying process in textile industries^6^. The excessive amount of dyes in industrial wastewater has posed a potential threat to the environment. These pollutants enter the food chain through aquatic organisms and get concentrated in human bodies^7,8^. Additionally, due to the high stability and non-biodegradability of dyes, they are often resistant to biological degradation^9^. Rose Bengal (RB) is a fluorescein dye, also known as Acid Red 94 used for a number of medical diagnostic procedures, such as liver function tests, staining of ocular necrotic tissue, and diagnosing conjunctivitis using devitalized corneal cells^10^. It damages mucous membranes when inhaled, making breathing uncomfortable for people. The chemical structure of RB is illustrated in Fig. 1. Therefore, scientists are continuously developing new ways to efficiently remove toxic dyes from wastewater.

In recent years, the advanced oxidation process (AOP) emerged as an effective technique for removing organic contaminants from wastewater^11^. Active species like hydroxyl radicals are generated by light, which starts the degradation reaction of organic compounds^12,13^. AOPs are gaining much interest in the research field as they are environment-friendly, safe, economical, and highly efficient in removing a wide range of organic contaminants^14^. Several photocatalysts, including a metal oxide semiconductor, are being investigated as catalysts for treating wastewater because they are safe and easily synthesized^15,16^. Cerium oxide (CeO_2_) is a common metal oxide semiconductor extensively explored for degrading pollutants from wastewater^17^. Ceria is an effective photocatalyst due to its high chemical resistance and oxygen-carrying capacity^18,19^. However, some limitations, like a wide bandgap between 2.6–3.2 eV, making it ineffective in visible light. Still, it is an excellent photocatalyst under the UV spectrum. Various metal sulfides have also been explored as efficient semiconductor photocatalysts owing to their larger surface area, low bandgap, and more exposed active sites^20–22^. Cadmium sulfide is a potential photosensitizer due to its low bandgap of 2.4 eV. It is effectively employed in various visible light-based catalytic materials like photovoltaic cells, optical electronics, and photocatalysts. Due to its lower conduction band gap edge, CeO_2_ effectively reacts with molecular oxygen to produce superoxide radicals, resulting in contaminants' degradation. The Ce^+3^ ions get oxidized to Ce^+4^ in the presence of H_2_O_2_ generating OH^·^ radicals which further initiate a series of chemical reactions producing reactive oxygen species (ROS) according to the reactions illustrated below:

1 $${\text{Ce}}^{ + 3} + {\text{H}}_{2} {\text{O}}_{2} \to {\text{Ce}}^{ + 4} + {\text{OH}}^{ - } + {\text{OH}}^{ \cdot }$$

2 $${\text{OH}}^{ \cdot } + {\text{H}}_{2} {\text{O}}_{2} \to {\text{H}}_{2} {\text{O}} + {\text{HO}}_{2}^{ \cdot }$$

3 $${\text{Ce}}^{ + 4} + {\text{HO}}_{2}^{ \cdot } \to {\text{Ce}}^{ + 3} + {\text{H}}^{ + } + {\text{O}}_{2}$$

While working with conventional iron-based Fenton reagents, the pH must be acidic for optimum efficiency; however, CeO_2_-based photocatalysts can operate at neutral pH, lowering operating costs^23^.

Several researchers have investigated the photocatalytic properties of CdS and CeO_2,_ but individually, these compounds do not possess adequate photocatalytic efficiency under visible light because of their larger bandgap and rapid charge recombination rate^24^. Some researchers examined binary and ternary nanocomposites of CdS and CeO_2_ for the removal of organic pollutants_,_ but they are effective only in aqueous solutions, and they are not very effective in removing highly concentrated dyes from wastewater. The photogenerated charge transfer via an internal electric field often might boost the photocatalytic activity in nanocomposites based on a solid p-n heterojunction interface^25,26^. One of the most efficient ways to encourage charge separation is using CeO_2_ and other semiconductors to form nanocomposites. This study has attempted to investigate the one-step synthesis of CdS/CeO_2_ heterojunction via a facile co-precipitation method and to investigate its photocatalytic performance for the photodegradation of concentrated dyes present in industrial effluents. The crystal structure and optical properties of the prepared composite were analyzed. The photocatalytic activity of the prepared nanocomposite was investigated for the removal of toxic dye RB from the aqueous stream. The deposition of CdS over the surface of CeO_2_ resulted in increased surface area and a greater number of active sites, thus enhancing the photocatalytic efficiency of the composite. The mechanism of degradation of RB over the surface of CdS/CeO_2_ under sunlight was investigated. The effect of hydrogen peroxide on the photocatalytic efficiency of the prepared composite is also reported.

## Experimentation

### Reagents

Cadmium chloride (CdCl_2_), Cerium nitrate hexahydrate (Ce(NO_3_)_2_.6H_2_O), Sodium sulfide (Na_2_S) flakes, sodium hydroxide (NaOH), Hydrogen peroxide (H_2_O_2_), dye–Rose Bengal, distilled water.

The above-mentioned chemicals, obtained from Sigma Aldrich, were of analytical grade and were used without further purification.

### Synthesis of CdS/CeO_2_ nanocomposite

**Figure 2:**
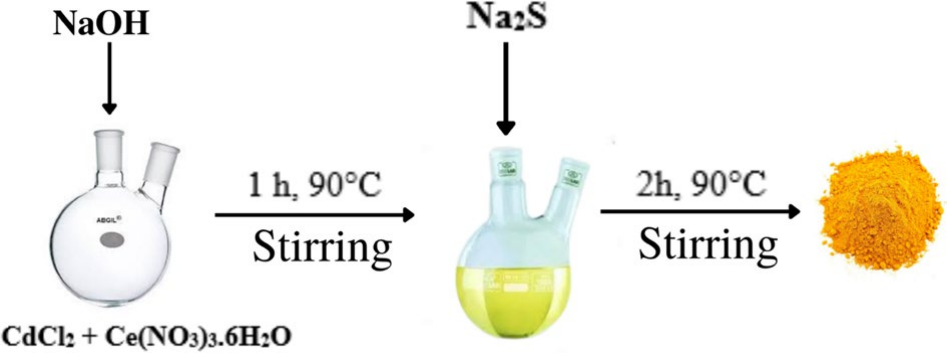
Synthesis process of CdS/CeO_2_ nanocomposite.

The CdS/CeO_2_ nanocomposite was synthesized via a one-pot co-precipitation method. Different amounts of CdCl_2_ and Ce(NO_3_)_3_.6H_2_O were taken in a doubled-necked round bottom flask to maintain a CdS:CeO_2_ ratio of 1:1, 1:2, 1:3, 2:1, and 3:1, dissolved in a minimum amount of distilled water and added a few drops of dilute NaOH. The solution was stirred for 1 h at 90 °C. The nitrate ions react with water molecules generating OH^-^ ions (Eq. 4) which further react with Ce^+3^ ions resulting in the formation of Ce(OH)_3,_ which is highly unstable and converted into CeO_2_ immediately (Eq. 5). Then added 15% 30 mL of Na_2_S dropwise, an instant source of S^2−^ ions. Due to a low solubility product of cadmium sulfide, CdS is precipitated (Eq. 6) over the surface of CeO_2_. Furthermore, CdS and CeO_2_ can be precipitated easily due to a low solubility product of CeO_2_ (K_sp_ = 7 × 10^−21^) and CdS (K_sp_ = 8 × 10^−27^) compared to Ce(OH)_3_ (K_sp_ = 1.5 × 10^−20^), Ce_2_S_3_ (K_sp_ = 4.4 × 10^−20^) and Cd(OH)_2_ (K_sp_ = 2.5 × 10^−14^)^27^. After adding sodium sulfide, the reaction was continued at the same temperature for another 2 h and then allowed to cool at room temperature. The final product was centrifugated several times, washed with distilled water and ethanol, and dried at 50 °C for 2 h. A schematic illustration of the synthesis process of CdS/CeO_2_ nanocomposite is presented in Fig. 2. Pristine CdS was synthesized by the same method with some slight modifications. Initially, 1 mmol of CdCl_2_ was dissolved in a minimum amount of distilled water for 15 min at room temperature and then CdS nanoparticles were precipitated by adding 15% 30 mL Na_2_S solution. The resulting yellow precipitate was stirred for 2 h, washed with distilled water several times, and dried at 50 °C for 2 h. For the synthesis of pristine CeO_2_ nanoparticles, 1 mmol of Ce(NO_3_)_3_·6H_2_O and 3 mmol of NaOH were dissolved separately in 20 mL of distilled water. The NaOH solution was added dropwise to the cerium salt solution, and the resulting purple solution was stirred at 90 °C for 3 h. The pale white precipitate was collected, washed with distilled water, and dried at 50 °C for 2 h.

4 $${\text{NO}}_{3}^{ - } + {\text{H}}_{2} {\text{O}} + {\text{e}}^{ - } \to {\text{NO}}_{2}^{ - } + 2{\text{OH}}^{ - }$$

5 $$4{\text{Ce}}^{3 + } + 12{\text{OH}}^{ - } + {\text{O}}_{2} \to 4{\text{CeO}}_{2} + 6{\text{H}}_{2} {\text{O}}$$

6 $${\text{Cd}}^{2 + } + {\text{S}}^{2 - } \to {\text{CdS}}$$

### Characterization

The crystal phase of the prepared sample was characterized by powder X-ray diffraction using Phillips X’Pert Pro diffractometer with Cu Kα radiations of wavelength 1.54056 Å and a scan speed of 2°/min at room temperature. The microstructure and surface morphology of the sample were analyzed using a high-resolution transmission electron microscope (JEM-2100). The elemental composition of CdS/CeO_2_ nanocomposite was analyzed by X-ray photoelectron spectroscopy (XPS) using AXIS ULTRA X-ray photoelectron spectrometer. The specific surface area and the pore structure of the sample were evaluated from nitrogen adsorption and desorption isotherms recorded by Quanta Chrome Nova 1000 gas adsorption analyzer. An FEI QUANTA FEG 200 high-resolution scanning electron microscope was used to record the SEM and EDAX. A Varian Cary eclipse fluorescence spectrophotometer was used to measure the PL intensities. All the UV adsorption studies were performed in Genesys 10S UV–vis spectrophotometer in 1 cm quartz cell from 400 to 800 nm wavelength at a speed of 600 nm-min.

### Assessment of photocatalytic activity

The photocatalytic activity of the CdS/CeO_2_ nanocomposite has been investigated by monitoring the degradation of Rose Bengal (RB) dye under solar irradiation. All the photocatalytic tests have been performed in 100 mL glass beakers under natural sunlight. The reaction mixture was stirred in the dark for 30 min to attain adsorption–desorption equilibrium, then kept in sunlight for 60 min. The photon lux was calculated using HTC LX-101 A luxmeter and found to be between 84,000–92,000 lx between 11 AM to 1 PM. The average radiation intensity at a reaction mixture’s surface was thus found to be 695.2 W/m^2^. The average reaction temperature was 32 °C. The progress of degradation was monitored by withdrawing the suspension solution for 10 min specific time intervals and taking the maximum absorbance of RB (544 nm).

The degradation efficiency (%) was calculated by the following equation:

7 $$Degradation\;Efficiency \left( \% \right) = \left( {\frac{{I_{C} - I_{t} }}{{I_{C} }}} \right) \times 100$$

where I_c_ (ppm) and I_t_ (ppm) are the initial concentration and concentration at time t of RB.

The kinetics of the photodegradation process was evaluated using the following equation:

8 $$\ln \frac{{I_{t} }}{{I_{C} }} = - kt$$

where I_c_ and I_t_ are concentration (ppm) at time t = 0 and t = t and k is the pseudo-first-order rate constant.

## Results and discussion

### Characterization

#### X-ray diffraction analysis

The structural properties and phase compositions of the synthesized nanocomposites with different molar ratios of CdS:CeO_2_ were analyzed by X-ray diffraction analysis using Philips X'PERT with Cu-Kα radiation having a scan speed of 2°/min at 25 °C.

**Figure 3:**
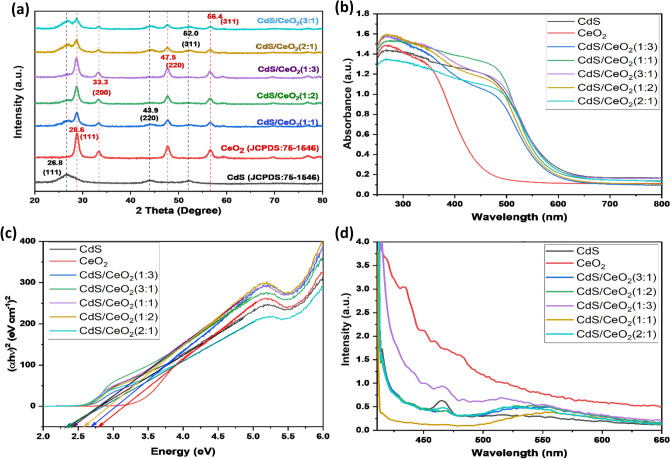
(**a**) XRD spectra, (**b**) UV–visible spectra, (**c**) Tauc’s plot, and (**d**) PL spectra of CdS, CeO_2,_ and CdS/CeO_2_ nanocomposites with different molar ratios.

The X-ray diffraction pattern of the synthesized CdS/CeO_2_ nanocomposites is represented in Fig. 3a. The peaks occurring at 2θ = 26.8_,_ 43.9, and 52.0, in the XRD spectrum of pristine CdS, could be indexed to the (111), (220), and (311), planes of hexagonal CdS (compared with JCPDS Card No 75-1546) while the peaks occurring at 2θ = 28.6, 33.3, 47.5, 56.4, 59.1, 69.4, 76.9, and 79.1 in the XRD spectra of CeO_2_ can be indexed to the (111), (200), (220), (311), (222), (400), (331) and (420) planes of the cubic structure of CeO_2_ (compared with JCPDS Card No 65-2975). The low intensity and broad peaks of CdS were attributed to low crystallinity and the small size of CdS nanoparticles. The XRD pattern of all the nanocomposites exhibited all the characteristic peaks of CeO_2_ with additional distinct peaks related to CdS, indicating CdS/CeO2 nanocomposite formation. No other peaks in the XRD spectrum of CdS and CeO_2_ could be seen, suggesting the high purity of the pristine samples. As seen in Fig. 3, as the molar ratio of CeO_2_ increases, the intensity of the peak at 2θ = 28.6° of CeO_2_ increases gradually at the same time, the intensity of the peak at 2θ = 28.6° of CdS decreases. Similarly, for the composites with increased CdS ratio, the intensity of the peak at 2θ = 28.6° of CdS also increases, indicating successful fabrication of CdS/CeO_2_ nanocomposites.

The average crystallite size of CdS/CeO_2_(1:1) nanocomposite was calculated by using Debye–Scherrer’s equation,

9 $$D = \frac{k\lambda }{{\beta cos\theta }}$$

where D is the crystallite size in nm, k is the shape factor (0.89), λ is the wavelength of *Cu-K*α radiation (λ = 1.54056 Å), β is full width at half maximum (FWHM) of the particular peak, and θ is the Bragg's angle. The average crystallite size was calculated to be 8.9 nm.

#### Optical properties

The optical response of CdS, CeO_2_, and CdS/CeO_2_ nanocomposite with different molar ratios was examined by UV–Vis diffuse reflectance spectroscopy (UV-DRS), as shown in Fig. 3b. The absorption spectra of CdS showed a broad absorption band ranging from about 260–500 nm with an absorption edge at 600 nm, while pristine CeO_2_ showed a narrow spectrum in the UV range with an absorption edge at 450 nm, reflecting a high bandgap of CeO_2_. All the prepared CdS/CeO_2_ nanocomposites with different molar ratios showed a similarly broad spectrum in the UV–visible range, from around 260 nm and extending to 500 nm, indicating outstanding light harvesting properties of the prepared photocatalyst. It is worth mentioning that no obvious change in the absorption spectra of CdS/CeO_2_ nanocomposites with different molar ratios was observed, indicating that CdS is grown over the surface of CeO_2_ and not incorporated within the lattice^28^. Furthermore, a decrease in the absorption edge with increasing CeO_2_ content signifies that the light absorption tendency of the nanocomposite is reduced as CeO_2_ can only absorb UV light^29^. The bandgap of the prepared nanocomposite was calculated using the Mott equation, i.e. αhυ ∝ (hυ-E_g_)^2^^30^, where α is the absorption coefficient, h is the Plank constant, and υ is the wavenumber. Regarding Tauc’s plot (inset Fig. 3c), the bandgap of CeO_2_ was calculated to be 2.8 eV, while CdS showed a comparatively low bandgap of 2.3 eV, indicating good visible light absorption properties^31^. The bandgap was slightly reduced for the CdS/CeO_2_ nanocomposites, as all the samples showed a comparable bandgap from 2.4 for a (1:1) ratio to 2.6 eV for a (1:3) molar ratio. The UV-DRS analysis suggests that the CdS/CeO2 nanocomposite bandgap could be tuned by adjusting the molar ratio of CdS/CeO_2_. The incorporation of CdS with CeO_2_ could reduce the bandgap and improve the photocatalytic activity of the nanocomposite in the visible region.

Photoluminescence (PL) spectroscopy is an important technique to access the visible light activity of the prepared nanocomposites. Pristine CeO_2_ showed the highest intensity in the PL spectrum suggesting the highest charge recombination rate of charges (Fig. 3d). The intensity of the PL peak decreases with the introduction of CdS with CeO_2_. The decrease in the PL intensity suggests that the charge recombination rate was significantly reduced, which is necessary for higher photocatalytic activity^22^. The CdS/CeO_2_(1:1) nanocomposite showed the lowest intensity in the PL spectrum, indicating that the charge recombination is slowest and was expected to show the highest photocatalytic activity.

#### Morphological studies

**Figure 4:**
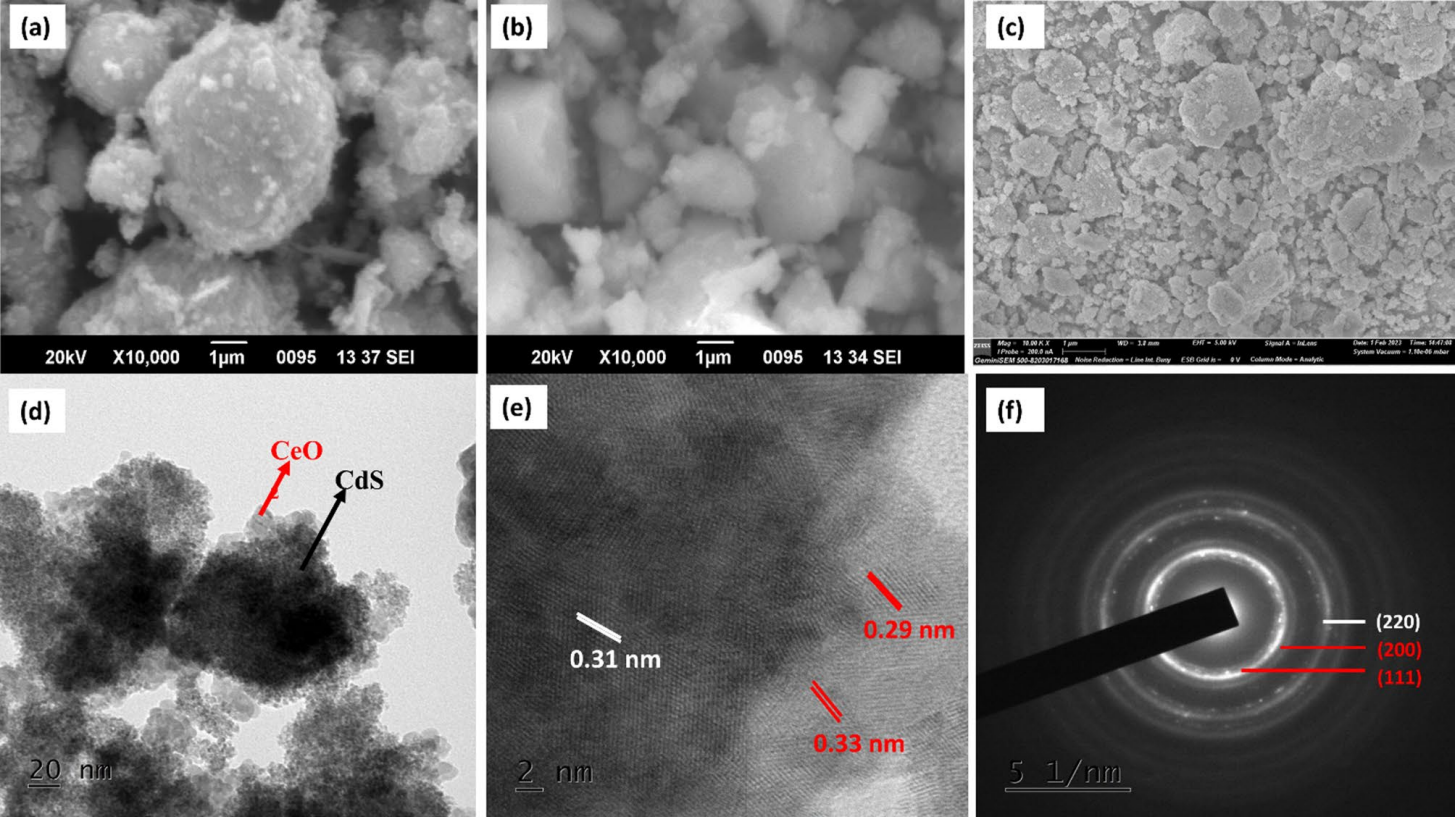
SEM image of (**a**) pure CdS, (**b**) pure CeO_2_ and (**c**) CdS/CeO_2_(1:1), (**d**) TEM micrograph, (**e**) HR-TEM micrograph and (**f**) SAED pattern CdS/CeO_2_(1:1) nanocomposite.

**Figure 5:**
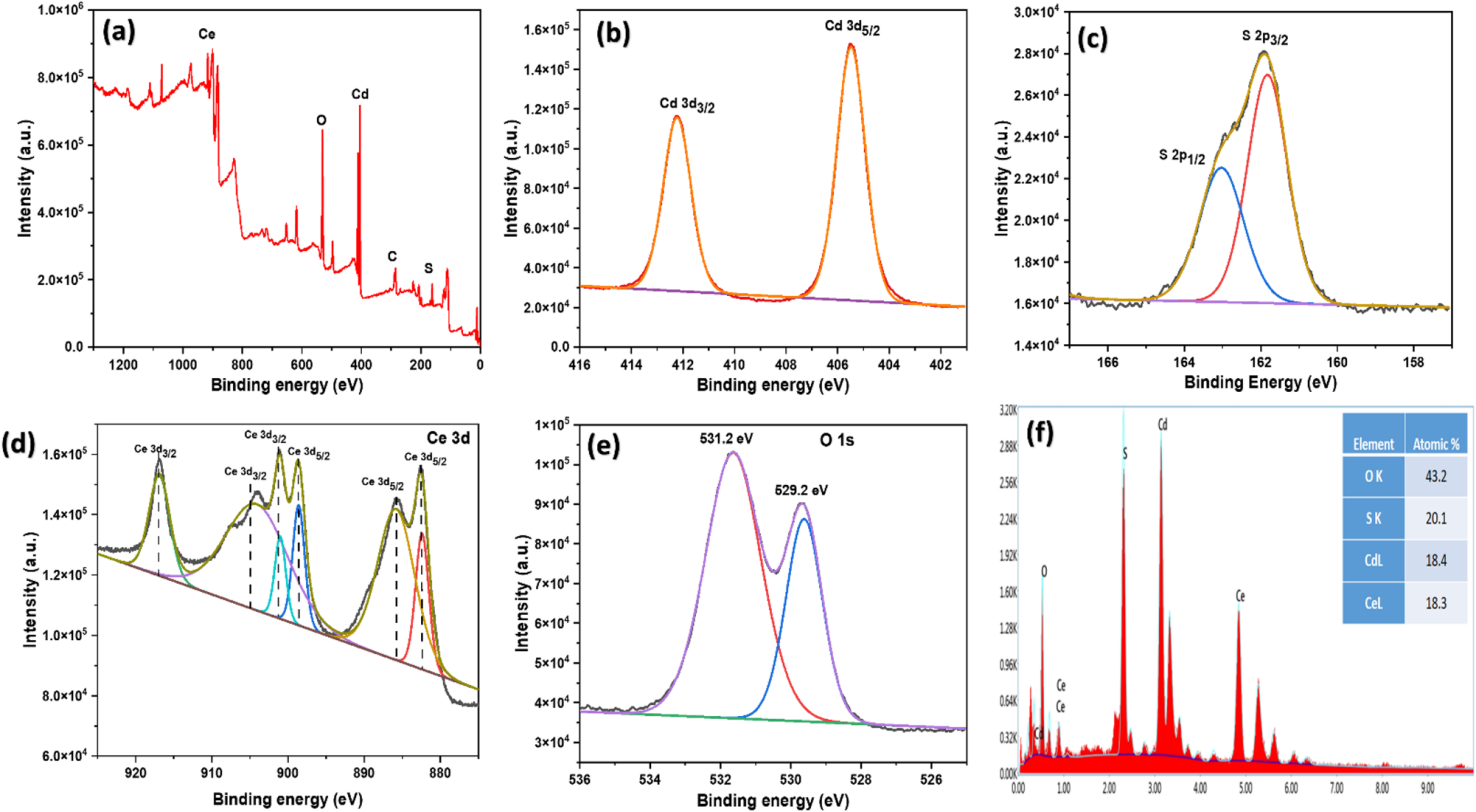
(**a**) Survey spectrum of CdS/CeO_2_(1:1), XPS narrow scan of (**b**) Cd 3*d*, (**c**) S 2*p*, (**d**) Ce 3*d*, and (**e**) O 1*s*, (**f**) EDAX spectra of CdS/CeO_2_(1:1) nanocomposite.

The surface morphology of the synthesized CdS/CeO_2_(1:1) nanocomposite was investigated using SEM. As illustrated in Fig. 4a,b, pure CdS nanoparticles showed spherical morphology, which could also be seen in TEM images; on the other hand, pure CeO_2_ showed small irregular cubic particles. The SEM images of CdS/CeO_2_(1:1) nanocomposite showed agglomeration of CdS nanospheres over the CeO_2_ surface (Fig. 4c). The TEM micrograph also showed the agglomeration of CdS nanoparticles over the cubic CeO_2_ surface. Two types of morphologies could be seen in the TEM images. On closer inspection, the TEM micrograph (Fig. 4d) revealed the inner part consists of the cubic CeO_2_ nanoparticles, and the outer greyish part is the CdS nanoparticles^32^. The high-resolution TEM (Fig. 4e) showed two types of interplanar lattice spacing on the surface, 0.33 nm, and 0.29 nm, which corresponds to the (111) and (200) planes of CdS, while the inner part showed consistent lattice fringes of 0.31 nm corresponding to (111) plane of CeO_2_. Furthermore, the formation of heterojunction between CdS and CeO_2_ can be clearly seen in Fig. 5c. Thus, TEM studies confirm the formation of CdS/CeO_2_ structure where CdS nanoparticles were anchored over the surface of CeO_2_. Due to the addition of NaOH, first CeO_2_ nanoparticles were formed, and after the addition of Na_2_S solution, CdS nanoparticles were precipitated over the surface of CeO_2_. The bright and concentric rings of the SAED pattern revealed the high crystallinity and polycrystalline nature of the synthesized CdS/CeO_2_(1:1) nanocomposite material, and the lattice planes (220) of CeO_2_ and (111) and (200) planes of CdS were identified and marked (Fig. 4f).

#### XPS analysis

**Figure 6:**
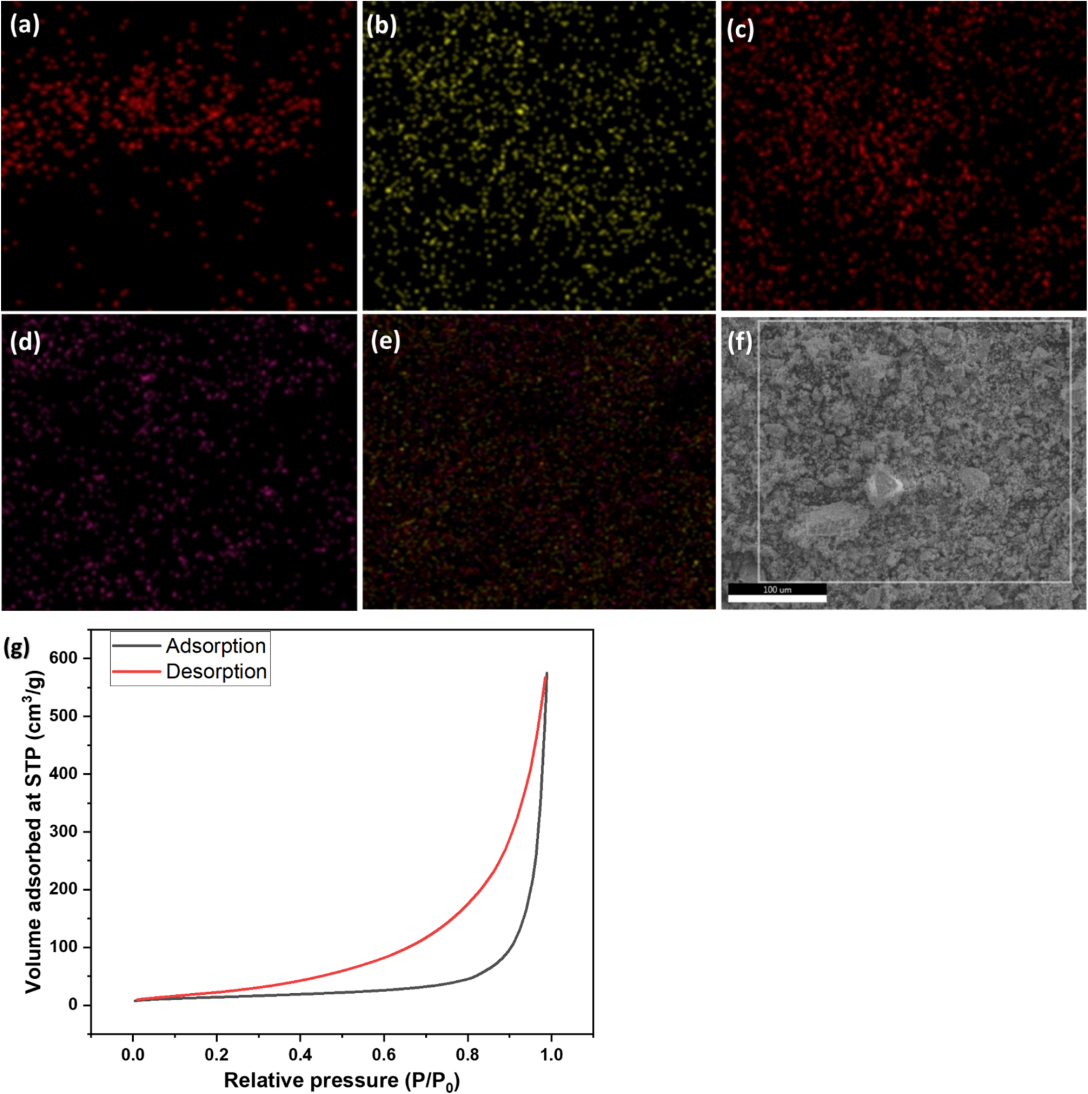
(**a**) Elemental mapping of (**a**) oxygen, (**b**) sulfur, (**c**) cadmium, (**d**) cerium (**e**) overall spectra, (**f**) SEM image for elemental mapping and (**g**) Nitrogen adsorption/desorption isotherm of CdS/CeO_2_(1:1) nanocomposite.

The elemental composition and oxidation states of metals were determined by XPS analysis of the prepared nanocomposite. The XPS survey spectrum (Fig. 5a) demonstrated peaks corresponding to Ce, Cd, O, S and C, and no impurity was found, confirming the samples' composition and high purity. The high-resolution XPS survey spectrum of Cd 3*d* and S 2*p* (Fig. 5b,c) showed two broad peaks at binding energies 411.6 eV and 405.1 eV for Cd and 168.2 eV and 161.5 eV for S, which can be indexed to Cd 3*d*_3/2_, Cd 3*d*_5/2_, S 2*p*_1/2,_ and S 2*p*_3/2_ respectively belonging to Cd^+2^ and S^−2^ oxidation states in CdS. The oxidation state of cerium oxide is an important parameter in determining its structure. The tetravalent Ce^+4^ in CeO_2_ is in a cubic fluorite structure^33^. The Ce 3d level consisted of several peaks centered at 916.4 eV, 906.8 eV, 900.6 eV, 898.2 eV, 885.2 eV, and 882.2 eV, respectively (Fig. 5d). It was reported that peaks at 882.2 eV, 898.2 eV, 906.8 eV, and 916.4 eV are related to the Ce^+4^ oxidation state, and the peaks at 885.2 eV and 900.6 eV are characteristics of Ce^+3^ oxidation state^34^. According to Fabris et al.^35^, partially reduced mixed-phase ceria is an intermediate phase. The presence of Ce^+3^ could be due to the presence of oxygen defects or a small amount of Ce_2_O_3_ in the sample^28^. This indicates the presence of both Ce^+3^ and Ce^+4^ ions in CdS/CeO_2_(1:1) nanocomposite, which further enhances the photocatalytic activity of the prepared heterojunction via a Fenton-like AOP system^23^. The deconvolution of O 1*s* peak (Fig. 5e) resulted in two distinct peaks at 529.2 eV and 531.2 eV, which can be attributed to adsorbed oxygen on the surface and lattice oxygen (O_L_) related to Ce^+4^ O^−^, respectively^36^. The higher intensity peak of lattice oxygen indicates high reactivity and lability of lattice oxygen in CeO_2_^37^. Furthermore, the electrons left behind by liable oxygen can be captured by Ce^+4^ ions and get reduced to Ce^+3^, which confirms the existence of Ce^+3^ ions in the Ce 3*d* spectrum^34^. Additionally, energy dispersive X-ray analysis (EDAX) analysis (Fig. 5f) of the prepared CdS/CeO_2_(1:1) nanocomposite revealed the presence of Cd, S, Ce, and O with respective atomic percentages of 16.16%, 18.71%, 19.20% and 45.93% which also confirms the successful fabrication of CdS/CeO_2_. The mapping images of the CdS/CeO_2_(1:1) (Fig. 6a–f) also revealed the presence of Cd, Ce, S, and O homogenously dispersed within the nanocomposite. Therefore, XPS, EDAX and mapping analysis confirms the successful fabrication of the CdS/CeO_2_(1:1) nanocomposite. The heterojunction between CeO_2_ and CdS results in the generation of highly reactive oxygen species, which improves the photocatalytic activity of the CdS/CeO_2_ nanohybrid.

#### Surface area analysis

The photocatalyst's surface area is a significant factor affecting photocatalytic activity. Generally, a larger surface area is desirable for an ideal photocatalyst. The Brunauer–Emmett–Teller (BET) analysis of CdS/CeO_2_(1:1) was carried out, and the specific surface area of the prepared composite was obtained as 51.30 m^2^/g with pore volume and pore diameter of 0.889 cm^3^/g and 693.00 Å, respectively, which could be due to agglomerated CdS nanoparticles^38^. Very high pore volume suggests that the prepared CdS/CeO_2_(1:1) nanocomposite is macroporous in nature. The results concluded that the photocatalytic activity is significantly affected by the surface area and pore size of the nanocomposite^1^.

Moreover, the nitrogen adsorption/desorption isotherm of the prepared composite showed hysteresis loops (Fig. 6g). The adsorption isotherm belongs to type IV isotherm, with the hysteresis loop having characteristics of H3 type for the prepared composite.

### Photocatalytic activity

#### Photocatalytic activity of different photocatalysts

**Figure 7:**
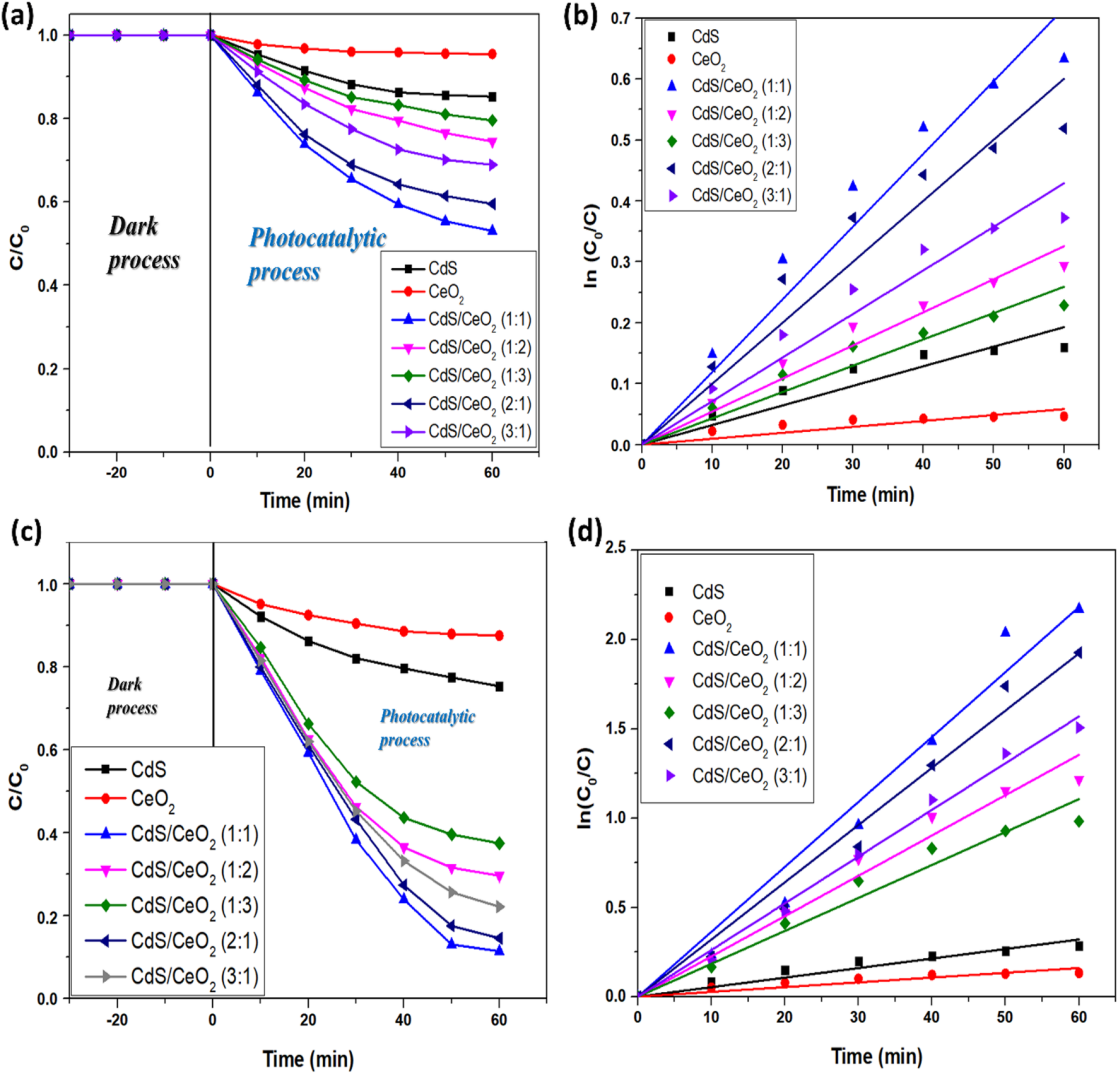
(**a**) Profiles of the degradation of RB dye, (**b**) degradation kinetics of different photocatalysts in absence of H_2_O_2_, (**c**) Profiles of the degradation of RB dye and (**d**) degradation kinetics of different photocatalysts in presence of H_2_O_2_.

The photocatalytic activity of different catalysts (2 mg) towards the degradation of 50 mL 150 ppm RB dye is shown in Fig. 7a,b. Although such high concentrations of dyes are usually not found in wastewater, higher dye concentrations are employed in photodegradation studies for better evaluation of the performance of the photocatalyst^39^. Before starting photodegradation, the dye solution with catalyst was stirred in the dark for 30 min to attain adsorption/desorption equilibrium. A blank experiment was done without a catalyst showing a negligible rate to rule out the possibility of self-photolysis of the dye. The characteristic UV–visible absorption peak of the dye decreased with time in the presence of the photocatalyst, which shows that the dye was degraded under solar irradiation by the photocatalyst.

The photodegradation efficiency of RB dye reached 46.92% for CdS/CeO_2_(1:1) nanocomposite whereas pristine CeO_2_ and CdS showed 4.55% and 14.75% degradation within 60 min. However, hydrogen peroxide is a well-known oxidizing agent commonly used in the photodegradation of organic compounds to enhance the photodegradation efficiency. Therefore, a small amount of hydrogen peroxide was added to boost the photodegradation and the amount of hydrogen peroxide was optimized further.

The photocatalytic activity of different catalysts towards the degradation of 50 mL 150 ppm RB dye in presence of 0.2 mL H_2_O_2_ is shown in Fig. 7c,d. The degradation efficiency of RB increased to 12.45% and 24.69% for pristine CeO_2_ and CdS, respectively. The results showed that pristine samples do not possess sufficient photocatalytic activity toward the degradation of RB dye. The reason could be due to a wide bandgap of CeO_2,_ which only absorbs UV light, while the bandgap of CdS corresponds to the visible region, but rapid recombination of charges limits its photocatalytic activity^17^. However, the photodegradation of RB dye was significantly improved, and a maximum degradation of 88.60% could be achieved within 60 min using CdS/CeO_2_(1:1) nanocomposite. The increased efficiency was attributed to the formation of heterojunction between CdS and CeO_2_ nanoparticles, which helps to delocalize the photoinduced electrons and holes, as also evident from the PL studies. Furthermore, the photogenerated electrons could react with adsorbed oxygen to produce superoxide radicals, and the holes could oxidize hydroxide ions to hydroxyl radicals in the CdS/CeO_2_(1:1) nanocomposite. We also observed that the photodegradation efficiency was affected by the ratio of CdS and CeO_2_. The best performance was shown by CdS/CeO2(1:1). This is because pure CeO_2_ could not produce sufficient reactive oxygen species. At the same time, a higher amount of CeO_2_ suppresses the light-absorbing tendency of CdS, reducing the number of photogenerated electrons to recombine with holes, thereby suppressing the charge transfer^40^. Therefore, CdS/CeO_2_(1:1) nanocomposite was chosen for further photocatalytic experiments. Table 1 illustrates the degradation performance of different photocatalysts.

**Table 1:** Photodegradation performance of different catalysts.

Catalyst	Degradation (%)	Rate constant (min^−1^)	R^2^
CeO_2_	12.45	0.00267	0.9565
CdS	24.69	0.00534	0.97557
CdS/CeO_2_(1:1)	88.6	0.03637	0.98983
CdS/CeO_2_(1:2)	70.37	0.02258	0.98810
CdS/CeO_2_(1:3)	62.55	0.01845	0.98686
CdS/CeO_2_(2:1)	85.44	0.03245	0.99190
CdS/CeO_2_(3:1)	77.85	0.02616	0.99715

#### Effect of hydrogen peroxide

**Figure 8:**
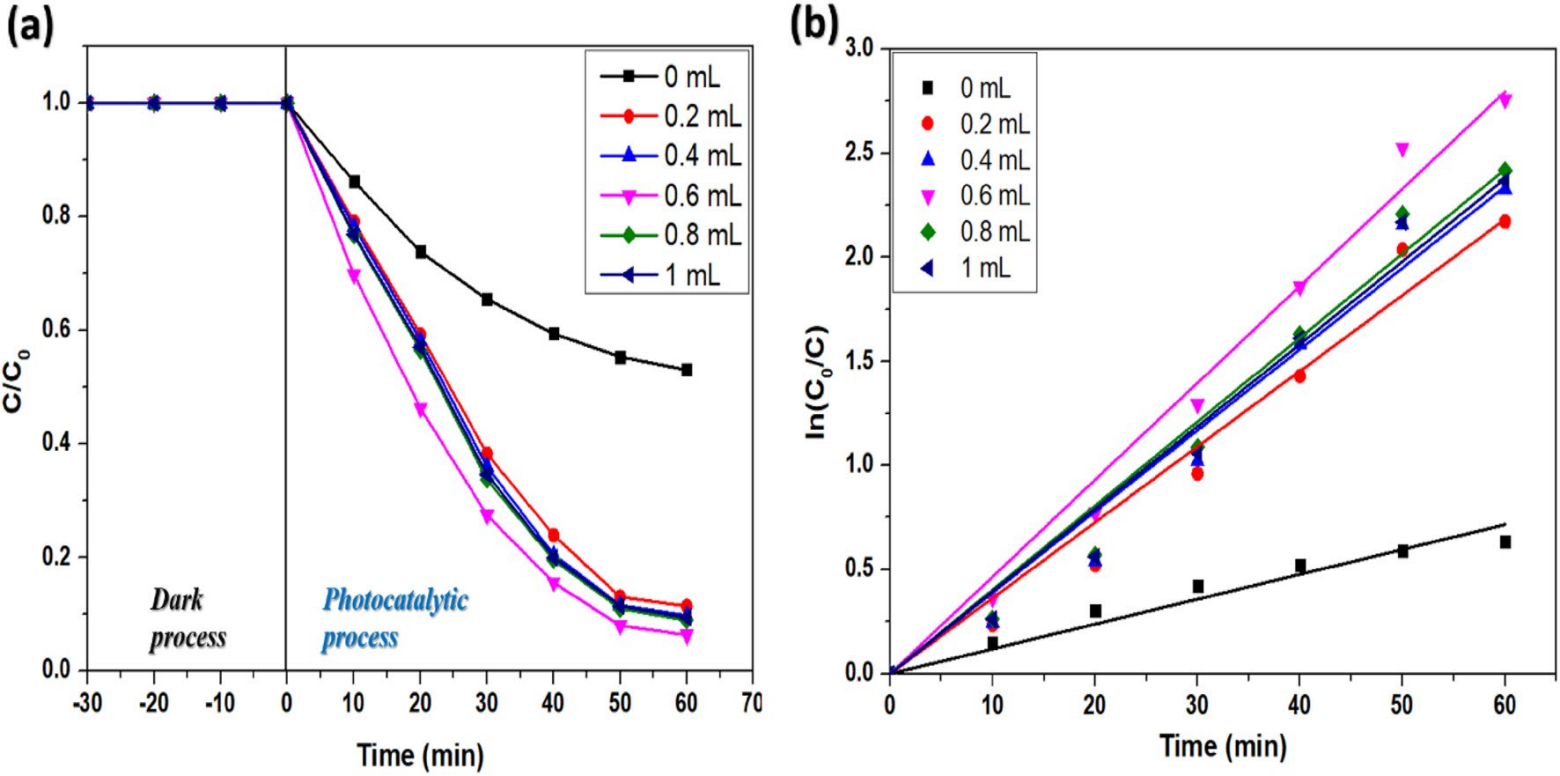
(**a**) Profiles of degradation of RB and (**b**) reaction kinetics at different H_2_O_2_ concentrations.

Hydrogen peroxide (H_2_O_2_) was reported to enhance the photocatalytic degradation of dyes in wastewater^41^. Therefore, the effect of the amount of hydrogen peroxide on the photocatalytic degradation efficiency of CdS/CeO_2_(1:1) nanocomposite was investigated by varying hydrogen peroxide amounts in the range of 0.2 mL/50 mL to 1 mL/50 mL of dye solution under sunlight irradiation. It is worth mentioning that no significant degradation (< 8%) occurred in presence of a catalyst and H_2_O_2_ under the dark suggesting the non-existence of the Fenton reaction in dark conditions. The photocatalytic activity of CdS/CeO_2_(1:1) was improved in presence of H_2_O_2_. The degradation efficiency increased with the amount of hydrogen peroxide to 0.6 mL, but a slight decrease in efficiency was observed beyond 0.6 mL. The degradation efficiency reached 93.65% within 60 min of irradiation in the presence of H_2_O_2_ (Fig. 8a), whereas only 46.92% of the dye could be degraded without peroxide in 60 min. The kinetics of photodegradation of RB in the presence of 0.6 mL hydrogen peroxide was examined (Fig. 8b). The effect of hydrogen peroxide on the degradation of RB and the reaction kinetics is illustrated in Table 2. The degradation process follows pseudo-first-order kinetics, and the rate constant was calculated to be 0.047 min^−1^. Furthermore, no significant decrease in RB concentration was observed with H_2_O_2_ without the photocatalyst ruling out the possibility of self-degradation of RB.

**Table 2:** Degradation of RB at different H_2_O_2_ dosages.

Dosage of H_2_O_2_ (mL/50 mL)	Degradation percentage	k (min^−1^)	R^2^
0	46.92	0.01284	0.99119
0.2	88.60	0.03649	0.98005
0.4	90.24	0.03913	0.97984
0.6	93.65	0.04700	0.99162
0.8	91.08	0.04043	0.98343
1.0	90.62	0.03973	0.98322

The increased photodegradation efficiency in the presence of H_2_O_2_ could be attributed to the production of a large number of hydroxyl radicals. The efficiency decreased beyond 0.6 mL because a higher peroxide concentration leads to the capture of hydroxyl radicals by H_2_O_2_ to form water and HO_2_^·^ radicals according to the following reactions^42,43^:

10 $${\text{H}}_{2} {\text{O}}_{2} + {\text{e}}^{ - } \to {\text{HO}}^{ \cdot } + {\text{HO}}^{ - }$$

11 $${\text{H}}_{{2}} {\text{O}}_{{2}} + {\text{O}}_{{2}}^{ \cdot - } \to {\text{HO}}^{ \cdot } + {\text{HO}}^{ - } + {\text{O}}_{{2}}$$

12 $${\text{H}}_{{2}} {\text{O}}_{{2}} + {\text{HO}}^{ - } \to {\text{H}}_{{2}} {\text{O}} + {\text{HO}}_{{2}}^{ \cdot }$$

13 $${\text{HO}}_{2}^{ \cdot } + {\text{HO}}^{ \cdot } \to {\text{H}}_{2} {\text{O}} + {\text{O}}_{2}$$

The reaction of H_2_O_2_ with free electrons and O_2_^·−^ yields additional hydroxyl radicals, which promote the degradation efficiency by increasing the amount of hydrogen peroxide according to Eqs. (10 and 11). Further increment in the amount of hydrogen peroxide causes a decrease in degradation efficiency because excess H_2_O_2_ traps hydroxyl radicals, generating HO_2_^·^ which does not play a major role in dye degradation. The trapping of hydroxyl radicals occurs according to Eqs. (12 and 13). A decrease in hydroxyl radicals due to increasing hydrogen peroxide dosage restricts the degradation of RB. Therefore, 0.6 mL H_2_O_2_/50 mL is fixed for further photocatalytic experiments.

#### Effect of pH

**Figure 9:**
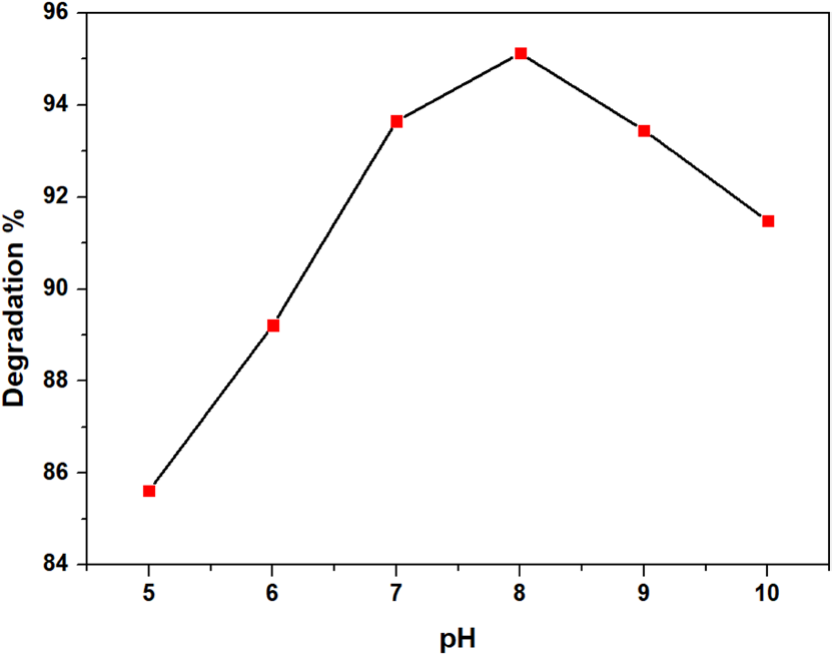
Effect of pH on the degradation of RB.

The pH of the solution is a significant factor affecting the photodegradation of dye in the aqueous phase^44^. The effect of pH on the degradation of RB was investigated by keeping the other parameters constant (Initial dye concentration = 150 ppm, Volume = 50 mL, Catalyst dosage = 2 mg and H_2_O_2_ dosage = 0.6 mL) and varying pH from 4 to 10. As illustrated in Fig. 9, the degradation of RB increases with an increase in pH, and about 95.12% degradation could be achieved at pH 8 within 60 min of solar irradiation. This could be explained by the fact that under alkaline conditions, more hydroxyl ions are present in the solution, which interact with positively charged holes generating more hydroxyl ions according to the following equation^45^:

14 $${\text{HO}}^{ - } + {\text{h}}^{ + } \to {\text{HO}}^{ \cdot }$$

However, at higher pH, more hydroxyl radicals are adsorbed on the photocatalyst surface; therefore, the surface of the photocatalyst becomes negatively charged, thereby inhibiting the generation of hydroxyl free radicals and causing repulsion between the photocatalyst and the dye molecules resulting in a decreased degradation efficiency^46^.

#### Effect of catalyst dosage

**Figure 10:**
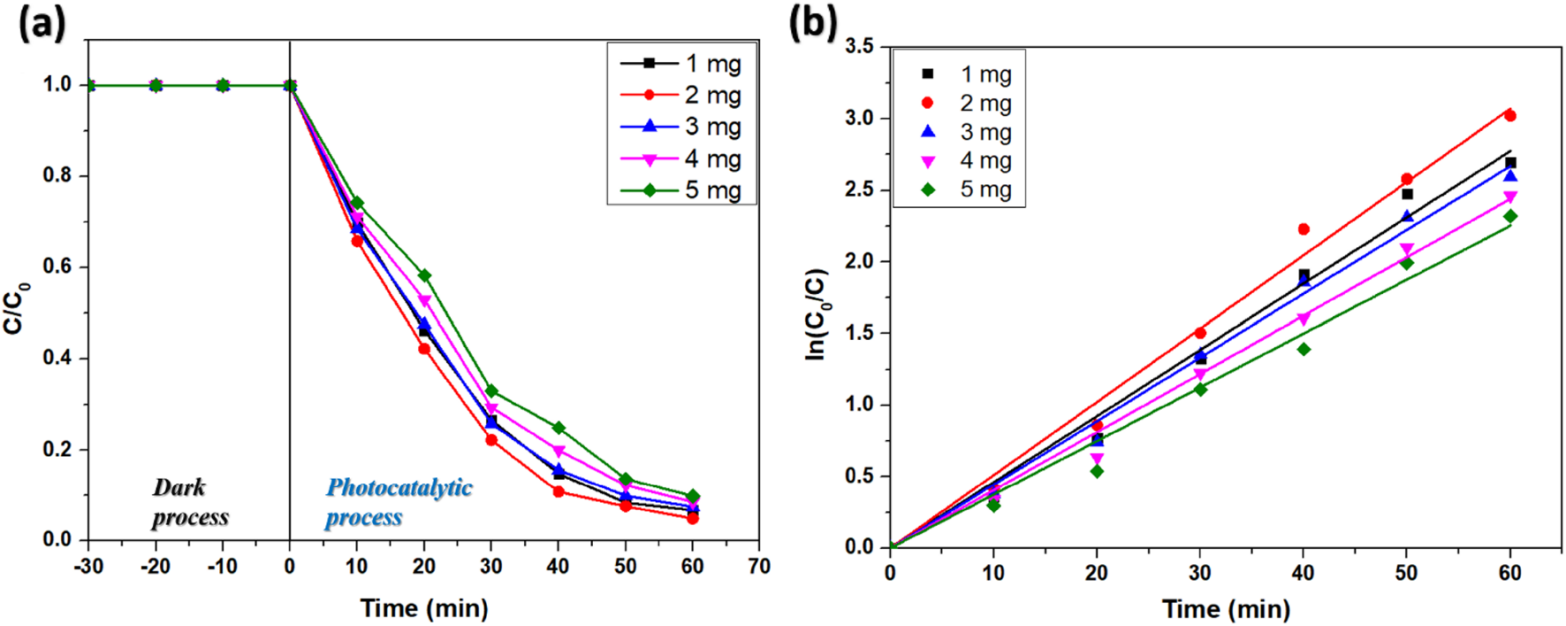
(**a**) Profiles of degradation of RB and (**b**) kinetics at different catalyst dosages.

The amount of photocatalyst dosage greatly influences the degradation of pollutants through AOP. To investigate the effect of catalyst concentration, other parameters like initial dye concentration, pH, and H_2_O_2_ dosage were fixed at 150 ppm, 8, and 0.6 mL, respectively, and the photocatalyst dosage was varied from 1 mg/50 mL to 5 mg/50 mL. The 2 mg/50 mL catalyst dosage showed 98.6% degradation of the dye solution within 60 min of irradiation with a pseudo-first-order rate constant of 0.05174 min^−1^ (Table 3; Fig. 10b). However, the degradation efficiency declined after that (Fig. 10a). The initial increase in the degradation percentage could be attributed to an increased number of active sites for the generation of ROS. However, at a higher catalyst dosage (> 2 mg) due to, the collision of nanoparticles in the solution phase blocks the sunlight from penetrating deep into the catalyst surface, decreasing the degradation of RB dye^47^. Hence the photocatalyst dosage was fixed at 0.04 g/L.

**Table 3:** Degradation of RB at varying photocatalyst dosage.

Catalyst Dosage (mg/50 mL)	Degradation percentage	k (min^−1^)	R^2^
1	93.25	0.04710	0.99362
2	95.12	0.05174	0.99656
3	92.52	0.04531	0.99412
4	91.48	0.04041	0.99465
5	90.18	0.03697	0.98839

#### Effect of initial dye concentration

**Figure 11:**
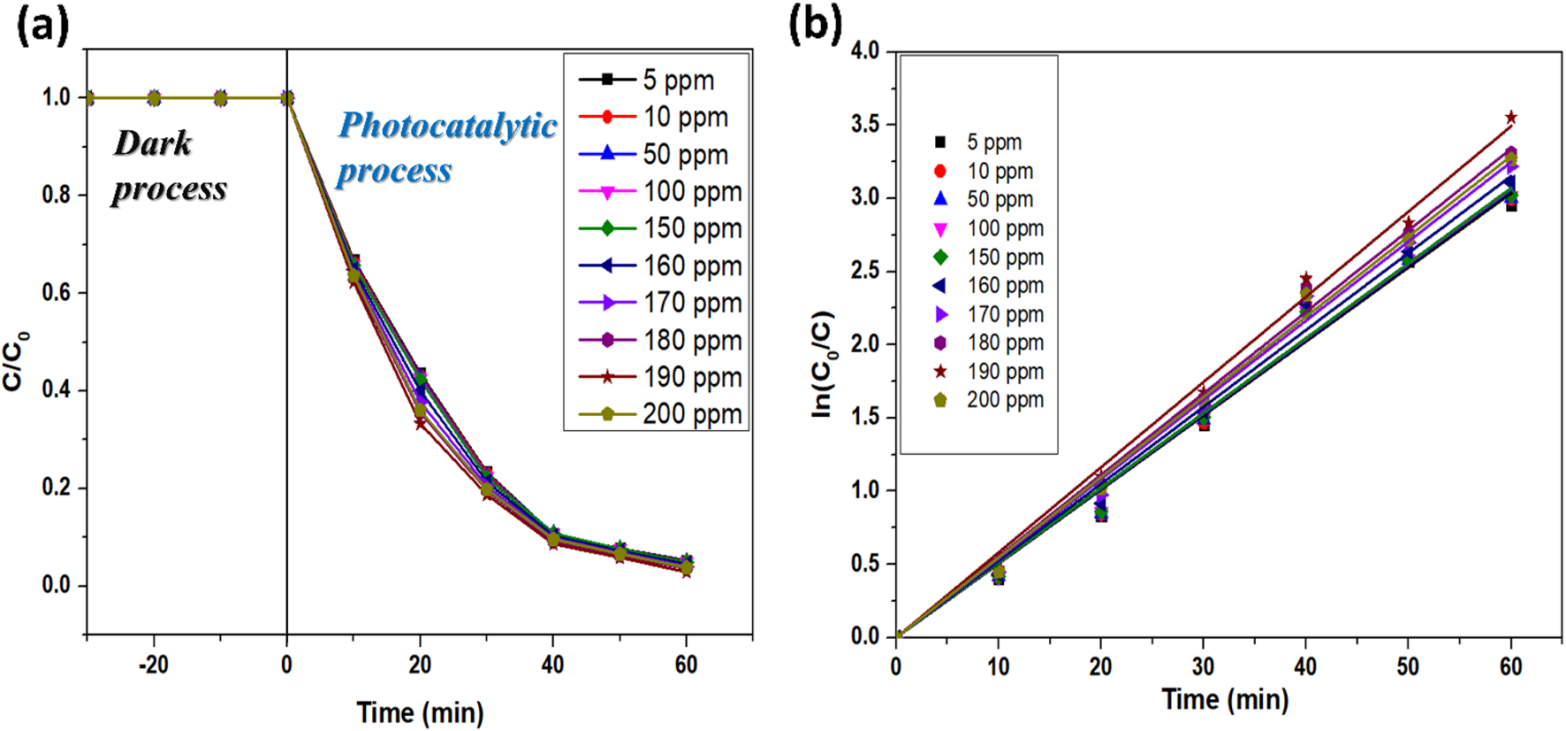
(**a**) Profiles of degradation of RB and (**b**) kinetics at different RB concentrations.

To further investigate the effect of the concentration of RB on the performance of the photocatalyst, the initial dye concentration was varied in the range of 5–200 ppm, keeping the other reaction parameters constant. No significant effect on the degradation performance of the photocatalyst was observed up to 190 ppm (Fig. 11a). After that, the degradation efficiency slumped to 96.2% for 200 ppm, which could be attributed to the hindrance in the path of photons due to higher dye concentration^48^. Furthermore, higher dye concentrations will require a greater photocatalyst loading which will further increase the opacity of the solution^49^. Studies claimed that increasing the dye concentration blocks the photocatalyst's surface active sites, inhibiting ROS generation, and thereby decreasing the degradation efficiency^50^. The maximum degradation efficiency of 97.14% for 190 ppm RB could be attained with a pseudo-first-order rate constant of 0.05824 min^−1^ within 60 min (Table 4; Fig. 11b). Therefore, 190 ppm RB dye was chosen as the optimum dye concentration for further photodegradation tests.

**Table 4:** Degradation of RB at different initial concentrations.

Dye concentration (ppm)	Degradation percentage	k (min^−1^)	R^2^
5	94.8	0.05061	0.99461
10	95.0	0.05104	0.99499
50	95.04	0.05113	0.99614
100	95.08	0.05119	0.99637
150	95.12	0.05174	0.99656
160	95.52	0.05257	0.99716
170	96.0	0.05416	0.99776
180	96.34	0.05567	0.99813
190	97.14	0.05824	0.99827
200	96.18	0.05485	0.99833

#### Effect of contact time and synergistic index

**Figure 12:**
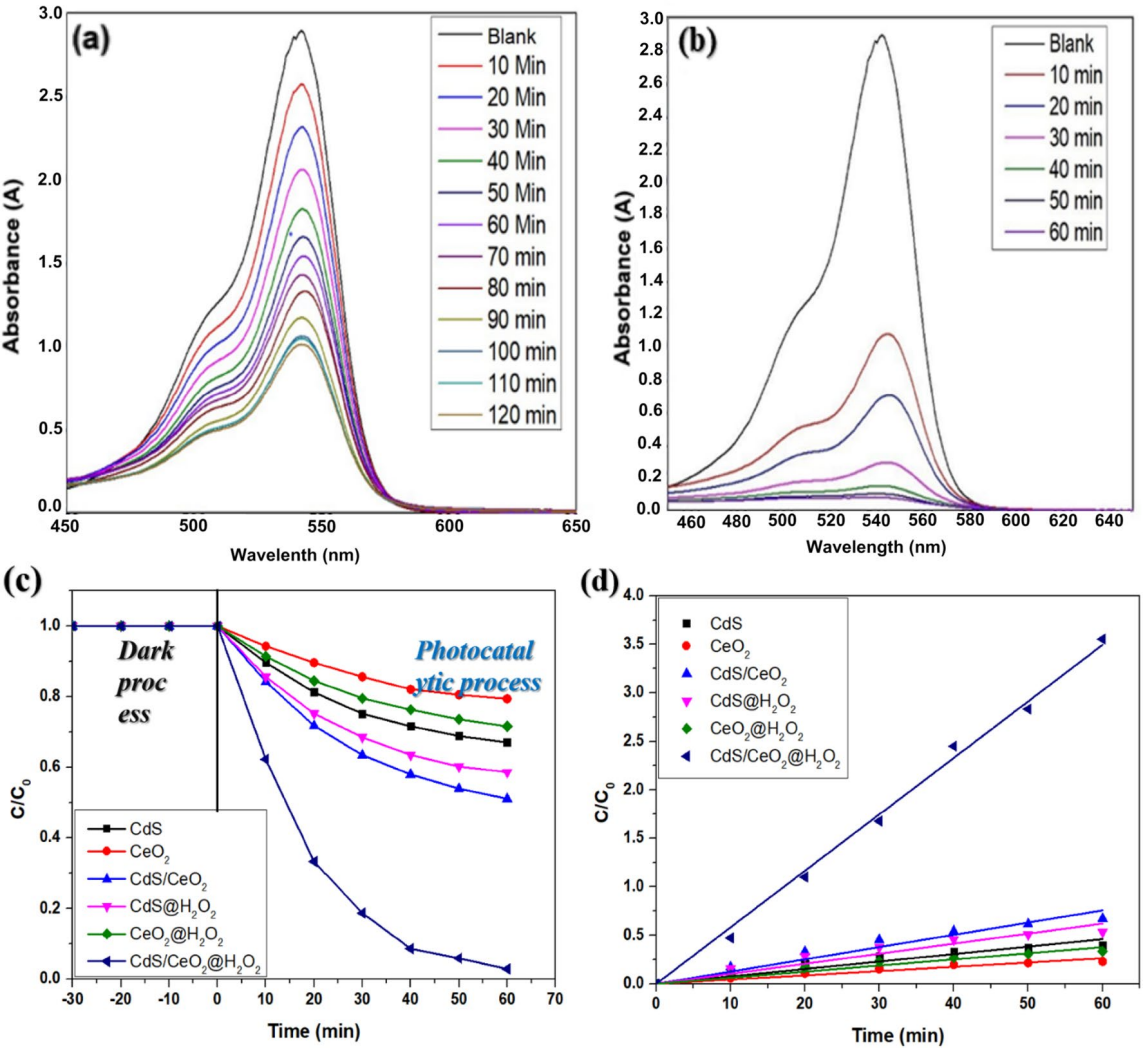
Effect of contact time on the degradation of RB (**a**) in the absence of H_2_O_2_ and (**b**) in the presence of H_2_O_2_, (**c**) Degradation profiles, and (**d**) kinetics of RB degradation by different catalysts in the presence and absence of H_2_O_2_.

Under optimum conditions of initial dye concentration of 190 ppm, 0.04 g/L photocatalyst dosage, 0.6 mL of H_2_O_2_ dosage, and at pH 8, no further increase in the degradation percentage was observed after 60 min of solar irradiation. This could be due to the exhaustion of the surface active sites of the photocatalyst. The highest degradation of 97.14% is reached under optimum conditions (Fig. 12b). Moreover, the degradation efficiency increases up to around 67.85% in the absence of H_2_O_2_ in 120 min, and no significant change was observed after that (Fig. 12a).

In order to study the synergistic effect between CdS and CeO_2_, the synergy index under optimum conditions in the presence and absence of H_2_O_2_ was calculated using the formula as follows^51^:

15 $${\text{Synergy}}\;{\text{index}} = \frac{{R_{{CdS/CeO_{2} }} }}{{R_{{CdS + R_{{CeO_{2} }} }} }}$$

where, ${R}_{CdS/{CeO}_{2}}$, ${R}_{CdS}$ and ${R}_{{CeO}_{2}}$ are the reaction rate constants of CdS/CeO_2_(1:1), CdS and CeO_2_, in absence of H_2_O_2_, respectively. Figure 12c,d illustrates the degradation performance of CdS, CeO_2,_ and CdS/CeO_2_(1:1) nanocomposite in the presence and absence of optimized amount of H_2_O_2_. As illustrated in Table 5, the reaction rate constants of ${R}_{CdS/{CeO}_{2}}$, ${R}_{CdS}$ and ${R}_{{CeO}_{2}}$ are 0.01262 min^−1^, 0.00767 min^−1^, and 0.00439 min^−1^, respectively, while the rate constants of the same in the presence of H_2_O_2_ are 0.05824 min^−1^, 0.0134 min^−1^, and 0.00630 min^−1^, respectively. The synergy index was calculated to be 1.0 in the absence of H_2_O_2_ and ~ 3.0 in the presence of H_2_O_2_, indicating that the combination of CdS and CeO_2_ and the formation of heterojunction is beneficial for H_2_O_2_ activation and the degradation of RB dye.

**Table 5:** Synergistic performance of different catalysts in the presence and absence of H_2_O_2_.

Catalyst	Degradation (%)		Rate constant (min^−1^)	
	In presence of H_2_O_2_	In absence of H_2_O_2_	In presence of H_2_O_2_	In absence of H_2_O_2_
CdS	41.34	32.93	0.0134	0.00767
CeO_2_	28.41	20.57	0.00630	0.00439
CdS/CeO_2_(1:1)	97.14	48.92	0.05824	0.01262

#### Scavengers test

**Figure 13:**
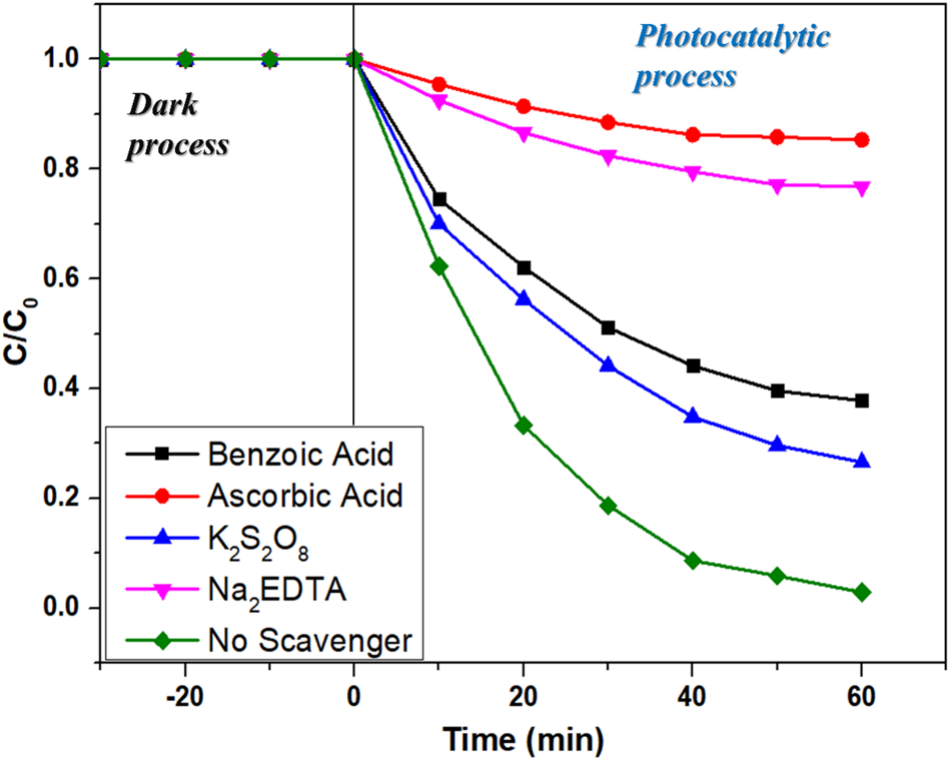
Profile of degradation of RB in presence of different scavengers.

The photodegradation reactions are controlled by the concentrations of ROS and the photogenerated charge carriers (electrons and holes) which generate them. Therefore, a few sacrificial agents were added to the reaction cell to trap the ROS and the charge carriers to investigate the effect of these radicals on the degradation of RB dye. Benzoic acid and ascorbic acid are used to trap OH^·^ and ^·^O_2_^−^ radicals. Potassium persulphate (K_2_S_2_O_8_) and Na_2_EDTA were employed at electron (e^−^) and hole (h^+^) scavengers. With the addition of 1 mmol of ascorbic acid and Na_2_EDTA, the degradation of RB slumped to 14.7% and 23.2%, respectively (Fig. 13). However, K_2_S_2_O_8_ and benzoic acid does not significantly affect the degradation process, indicating that the degradation of RB is mainly due to the presence of ^·^O_2_^−^ and h^+^^52^.

#### Mechanism of the degradation of RB

**Figure 14:**
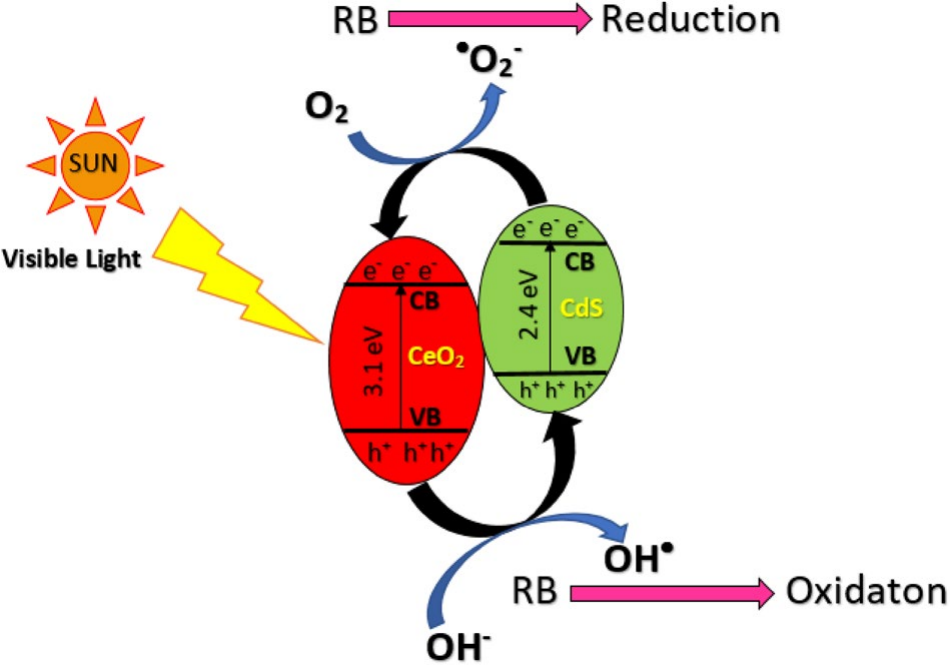
Mechanism of photocatalytic degradation of RB over CdS/CeO_2_(1:1) surface under solar irradiation.

Based on the above discussions, a schematic mechanism of photodegradation of RB over the CdS/CeO_2_(1:1) surface is shown in Fig. 14. When the fabricated photocatalyst is irradiated under sunlight, the electrons get excited from the valance band (VB) to the conduction band (CB) of CdS forming positively charged h^+^ in the VB due to its lower bandgap. The generated e^−^ immediately gets transferred to the CB of CeO_2_ and initiates a redox reaction forming a Ce^4+^/Ce^3+^ redox couple which in turn produces ROS contributing to the degradation of RB. Then these electrons interact with adsorbed oxygen generating ^·^O_2_^−^ radicals. The h^+^ in the VB of CeO_2_ moves to the VB of CdS, where they react with hydroxide ions generating OH^·^ radicals^53^. Finally, the RB dye molecules adsorbed on the surface of CdS/CeO_2_(1:1) get attacked by these ROS, resulting in its degradation. Furthermore, h^+^ in the VB also reacts with adsorbed RB initiating its degradation process. Thus coupling a low bandgap photocatalyst such as CdS with CeO_2_ proved to be beneficial for effective charge delocalization and improved photocatalytic activity. The steps in the photodegradation of organic pollutants by nanocomposite photocatalyst (NP) are illustrated below:

1. Photoexcitation of photocatalyst
  16 $${\text{NP}} + hv \to {\text{e}}^{ - } + {\text{h}}^{ + }$$
2. Ionosorption of adsorbed oxygen
  17 $${\text{O}}_{{2}} + {\text{e}}^{ - } \to {\text{O}}_{{2}}^{ \cdot - }$$
3. Followed by the ionization of water
  18 $${\text{H}}_{2} {\text{O}} \to {\text{HO}}^{ - } + {\text{H}}^{ + }$$
4. Oxidation of hydroxyl ions by h^+^
  19 $${\text{HO}}^{ - } + {\text{h}}^{ + } \to {\text{HO}}^{ \cdot }$$
5. Protonation of superoxide radical
  20 $${\text{O}}_{2}^{ \cdot - } + {\text{H}}^{ + } \to {\text{HO}}_{2}^{ \cdot }$$
6. Followed by co-scavenging of electron
  21 $${\text{HO}}_{2}^{ \cdot } + {\text{e}}^{ - } \to {\text{HO}}_{2}^{ - }$$
7. Formation of Hydrogen peroxide
  22 $${\text{HO}}_{{2}}^{ - } + {\text{H}}^{ + } \to {\text{H}}_{{2}} {\text{O}}_{{2}}$$
8. Followed by hydroxyl radical formation
  23 $${\text{H}}_{{2}} {\text{O}}_{{2}} + {\text{e}}^{ - } \to {\text{HO}}^{ \cdot } + {\text{HO}}^{ - }$$
9. Degradation of pollutants by active species
  24 $${\text{Pollutant}} + {\text{active}}\;{\text{species}}\;\left( {{\text{HO}}^{ \cdot } ,{\text{h}}^{ + } ,{\text{e}}^{ - } } \right) \to {\text{degradation}}\;{\text{products}}$$

### Reusability studies

**Figure 15:**
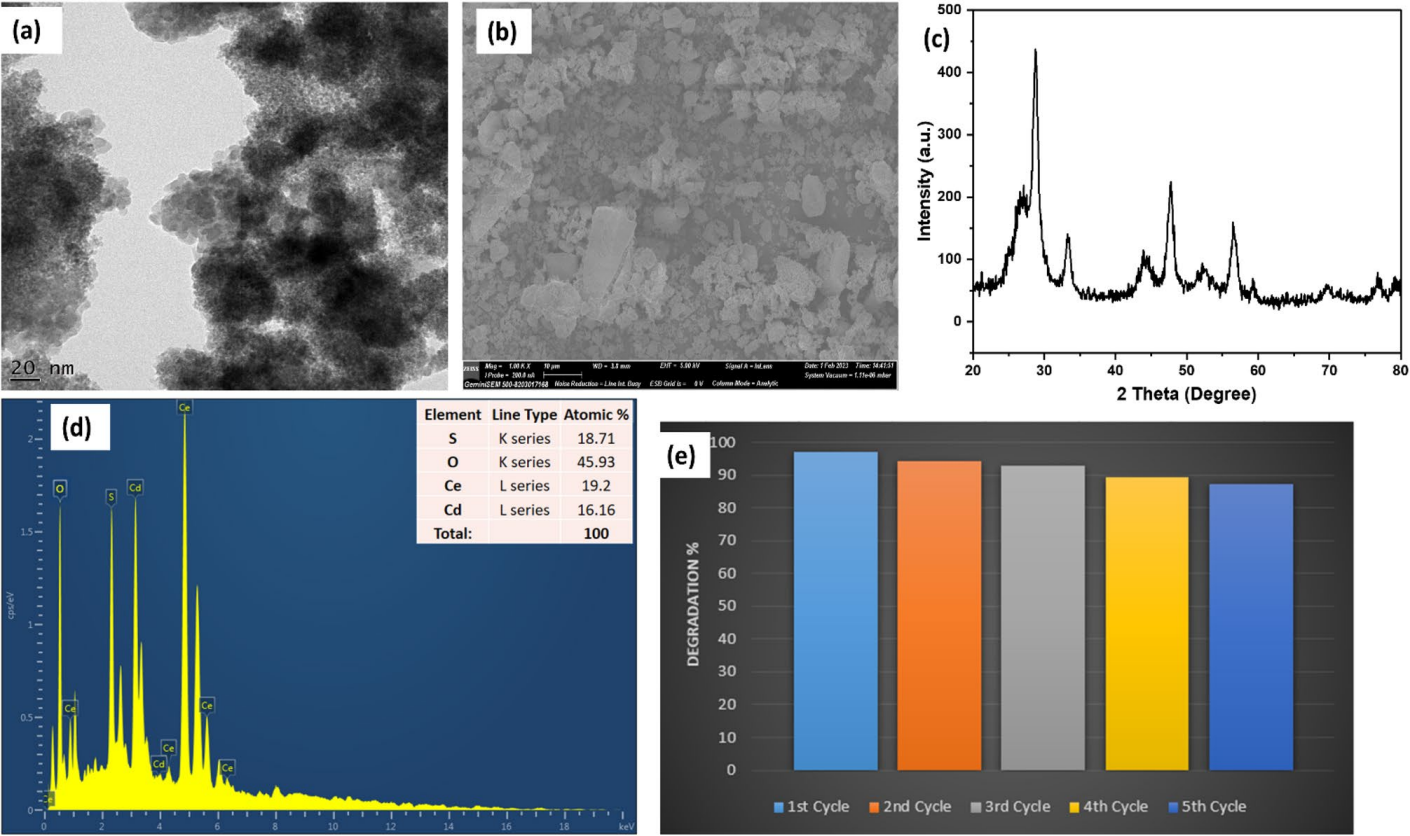
(**a**) TEM image, (**b**) SEM image, (**c**) EDAX spectra and (**d**) Reusability performance of CdS/CeO_2_(1:1) nanocomposite.

Reusability is an important factor in evaluating the stability and practical applications of the prepared sample. The as-prepared CdS/CeO_2_(1:1) nanocomposite was investigated for reusability by subjecting it to four consecutive experimental cycles under the same conditions. After each cycle, the sample was filtered, washed with ethanol and distilled water, and dried at 50 °C for 3 h before reusing for the next cycle. As illustrated in Fig. 15, the prepared photocatalyst shows a slight decrease in photocatalytic activity until the fourth cycle. The efficiency was almost the same in the next experiment. In the reusability experiment, the photocatalyst retained about 87% efficiency till the fifth cycle suggesting good stability and reusability of the as-prepared CdS/CeO_2_(1:1) nanocomposite. The reduction in the degradation efficiency might be due to the blockage of active sites by the degradation products during degradation^54^. Furthermore, the reused catalyst after the fifth cycle was characterized by XRD, EDAX, TEM, and SEM to evaluate its structural and chemical stability. As seen in Fig. 15c, the XRD spectrum showed all the peaks corresponding to CdS and CeO_2_ in the reused sample. The XRD suggests that the crystal structure of the reused catalyst was perfectly maintained after the fifth cycle. Additionally, the EDAX (Fig. 15d) spectrum confirms the presence of Cd, Ce, S, and O in the reused catalyst, suggesting that the CdS was not photo corroded during the photocatalytic tests. The formation of heterojunction nanocomposite effectively enhanced the charge separation which suppressed the photo corrosion of CdS^55^. Moreover, the ratio of Cd:S and Ce:O was maintained perfectly, suggesting excellent stability of the prepared photocatalyst. The TEM and SEM images (Fig. 15a,b) also revealed the presence of CeO_2_ and CdS in the reused nanocomposite, indicating that the prepared CdS/CeO_2_(1:1) nanocomposite maintained its structure after the fifth run.

## Conclusion

The one-step synthesis of CdS/CeO_2_(1:1) nanocomposite was carried out via the co-precipitation method, and the photocatalytic efficiency in the presence and absence of hydrogen peroxide was investigated. Various characterization techniques were used to assign the nanocomposite's structure, composition, and morphology. SEM, TEM, and HRTEM images revealed the accumulation of CdS nanoparticles over a cubic CeO_2_ surface. The nanocomposite photocatalyst showed excellent degradation efficiency of 97.14% within 60 min of solar irradiation in the presence of hydrogen peroxide with a pseudo-first-order rate constant of 0.05824 min^−1^. This is due to the formation of superoxide radicals and holes during the photocatalytic process. The prepared nanocomposite was highly stable and reusable for the photocatalytic degradation of Rose Bengal dye from wastewater, suggesting that the composite could be a potential photocatalyst for the degradation of textile dyes present in industrial effluents.

## Data Availability

All data generated or analyzed during this study are included in this published article.
